# The early response of oil palm (*Elaeis guineensis* Jacq.) plants to water deprivation: Expression analysis of miRNAs and their putative target genes, and similarities with the response to salinity stress

**DOI:** 10.3389/fpls.2022.970113

**Published:** 2022-09-23

**Authors:** Fernanda Ferreira Salgado, Thalliton Luiz Carvalho da Silva, Letícia Rios Vieira, Vivianny Nayse Belo Silva, André Pereira Leão, Marcos Mota do Carmo Costa, Roberto Coiti Togawa, Carlos Antônio Ferreira de Sousa, Priscila Grynberg, Manoel Teixeira Souza

**Affiliations:** ^1^Graduate Program of Plant Biotechnology, Federal University of Lavras, Lavras, MG, Brazil; ^2^The Brazilian Agricultural Research Corporation, Embrapa Agroenergy, Brasília, DF, Brazil; ^3^The Brazilian Agricultural Research Corporation, Embrapa Genetic Resources and Biotechnology, Brasília, DF, Brazil; ^4^The Brazilian Agricultural Research Corporation, Embrapa Mid-North, Teresina, PI, Brazil

**Keywords:** abiotic stress, tolerance, transcriptome, transcription factor, non-coding RNA, lncRNA

## Abstract

Oil palm (*Elaeis guineensis* Jacq.) is a oilseed crop of great economic importance drastically affected by abiotic stresses. MicroRNAs (miRNAs) play crucial roles in transcription and post-transcription regulation of gene expression, being essential molecules in the response of plants to abiotic stress. To better understand the molecular mechanisms behind the response of young oil palm plants to drought stress, this study reports on the prediction and characterization of miRNAs and their putative target genes in the apical leaf of plants subjected to 14 days of water deprivation. Then, the data from this study were compared to the data from a similar study that focused on salinity stress. Both, the drought-and salt-responsive miRNAs and their putative target genes underwent correlation analysis to identify similarities and dissimilarities among them. Among the 81 identified miRNAs, 29 are specific for oil palm, including two (egu-miR28ds and egu-miR29ds) new ones – described for the first time. As for the expression profile, 62 miRNAs were significantly differentially expressed under drought stress, being five up-regulated (miR396e, miR159b, miR529b, egu-miR19sds, and egu-miR29ds) and 57 down-regulated. Transcription factors, such as MYBs, HOXs, and NF-Ys, were predicted as putative miRNA-target genes in oil palm under water deprivation; making them the most predominant group of such genes. Finally, the correlation analysis study revealed a group of putative target genes with similar behavior under salt and drought stresses. Those genes that are upregulated by these two abiotic stresses encode lncRNAs and proteins linked to stress tolerance, stress memory, modulation of ROS signaling, and defense response regulation to abiotic and biotic stresses. In summary, this study provides molecular evidence for the possible involvement of miRNAs in the drought stress response in oil palm. Besides, it shows that, at the molecular level, there are many similarities in the response of young oil palm plants to these two abiotic stresses.

## Introduction

Oil palm (*Elaeis guineensis* Jacq.) is a palm tree from the Arecaceae family, classified as one of the most productive oil seed crops (Wang, L. et al., 2020). Originally from West Africa, it has been successfully introduced and exploited commercially in Asia, Africa, and Latin America. Indonesia and Malaysia are the largest palm oil-producing nations (EPOA, 2020). This species has great economic importance due to the high fruit production and oil efficiency extraction, with refining processes that render both palm oil and palm kernel oil (Corley, 2009; Silva et al., 2016). Palm oil is the raw material for cosmetics, medicines, candles, soaps, biofuels, and lubricating greases, and its demand is increasing (Abrapalma, 2018).

Oil palm does not withstand long periods of severe or moderate drought, and its fruit yield decreases considerably under water scarcity (Azzeme et al., 2016). It requires ~2,000 mm/year of water and does not tolerate drought for more than 90 days (Corley et al., 2018). According to Silva et al. (2016, 2017), water stress from seven to 21 days induced physiological changes and affected the growth of oil palm seedlings; while repetitive water deficit events induced photosynthetic acclimation in young oil palm plants (Lopes Filho et al., 2021). However, there is not much information about the molecular mechanisms behind the responses of oil palm plants to drought stress.

MicroRNAs (miRNAs) are small molecules of non-protein-coding RNAs, 20 to 24 nucleotides (nts) in length, derived from single-stranded precursors, which form a secondary stem-loop structure (Xin et al., 2015). They are involved in gene expression regulation at a post-transcription level in plants, animals, fungi, and viruses (Denli et al., 2004; Lytle et al., 2007; Ventura et al., 2008; Xu et al., 2019). miRNAs molecules are highly conserved in plants (da Silva et al., 2016).

Several studies have shown that miRNAs are involved in many biological and metabolic processes (Comai and Zhang, 2012; Sun, 2012). They play crucial roles in plant growth regulation (Mallory et al., 2004), flower development (Chen, 2004; Zhu et al., 2009), and responses to abiotic stresses, such as drought and salinity (Liu et al., 2008; Ding et al., 2009; Lv et al., 2010; Chen et al., 2018; Qiu et al., 2020; Salgado et al., 2021; Zeeshan et al., 2021).

Water stress-responsive miRNAs are present in *Oryza sativa* (Zhou et al., 2010), *Medicago truncatula* (Wang et al., 2011), and *Arabidopsis thaliana* (Liu et al., 2008). Zhou et al. (2010) described 11 down-and eight up-regulated miRNAs in rice plants under drought stress. Li et al. (2008) reported that miR169a and miR169c are significantly down-regulated by drought, promoting increased drought resistance in arabidopsis. They postulated that it was because one of the miR169 targets, NFYA5 (Nuclear Factor YA5), is a crucial transcription factor that regulates the expression of numerous drought stress-responsive genes (Li et al., 2008).

Studies have shown that miR398 and miR408 are positively regulated by water deficit in *M. truncatula*, leading to the negative regulation of its target genes (COX5b, CSD1, and plantacyanin; Trindade et al., 2010). In *Zea mays*, miRNAs modulate the expression of MAPK (mitogen-activated protein kinase), PLD (phospholipase D), PHD (proline dehydrogenase), and POD (peroxidase; Wei et al., 2009); which are known to be involved in plant response to environmental stresses as part of signaling pathways (MAPK and PLD) or as having role in the ROS-scavenging system (PHD and POD).

Although some studies identified miRNAs in oil palm (Md Nasaruddin et al., 2007; Xiao et al., 2013; Low et al., 2014; da Silva et al., 2016; Salgado et al., 2021), only a few miRNAs from *E. guineensis* are in miRBase (version 2.1). To the best of our knowledge, there are no reports on prospecting and characterizing drought-responsive miRNAs in oil palm. This study reports on the prediction and characterization of miRNAs responsive to drought stress in oil palm plants and their target genes. Besides, the drought- (from this study) and salt-responsive (from Salgado et al., 2021) miRNAs and their putative target genes underwent correlation analysis to identify similarities and dissimilarities among them.

## Materials and methods

### Plant material, growth conditions, experimental design, and drought stress

The oil palm plants used in this study were clones regenerated out of embryogenic calluses (Corrêa et al., 2015) obtained from leaves of an adult plant belonging to the *E. guineensis* genotype AM33, a Deli x Ghana from ASD Costa Rica.^1^ The plants used in this study were different of the one used in the Salgado et al. (2021) study; although, all plants – from both studies – came from the same embryogenic calluses; consequently, they are all clones of the same plant.

The embryogenic calluses were transferred to a regeneration medium on January 2016 and kept in a BOD chamber at 30°C and a 16/8-h light/dark photoperiod. On July and December 2016, the plants regenerated *in vitro* were put in 200-mL plastic cups containing vermiculite and a commercial substrate (Bioplant^®^ – Bioplant Agrícola Ltda., Nova Ponte, MG, Brazil), in a 1:1 ratio, on a dry basis; transferred to the PGW40 growth chamber (Conviron, Winnipeg, Canada), with air temperature at 25 ± 2°C, relative humidity at 60 ± 10%, and light intensity at 500 ± 50 μmol m^−2^ s^−1^, for acclimation; and then transferred to a greenhouse. Once acclimated, they were transferred to black plastic pots (3.5 L) containing 1,200 g of vermiculite, soil, and a commercial substrate (Bioplant^®^) mix, in a 1:1:1 ratio – on a dry basis; and fertilized using 2.5 g/L of the formula nitrogen (N), phosphorus (P), and potassium (K) fertilizer 20-20-20.

Before starting the experiments, plants were screened accordingly to their development stage, size, and number of leaves, to use the most uniform group of plants possible. The experiment was performed in a greenhouse in Brasília, DF, Brazil (S-15.732°, W-47.900°). Main environmental variables (temperature, humidity, and radiation), measured at a nearby meteorological station (S-15.789°, W-47.925°), fluctuated according to the weather conditions. Plants underwent drought stress in the growth stage known as bifid saplings.

Two experiments were carried out in November 2017 (Experiment 1) and March 2018 (Experiment 2), and both consisted of two treatments: a control one, with four replicates, and a drought stress one, with six replicates. The experimental design was completely randomized blocks. All plants in the control treatment were maintained at field capacity throughout the entire experiment, while plants in the drought stress treatment were initially at field capacity, and then they were deprived of water addition for 14 days.

### Evapotranspiration rate and gas exchange measurements

Plant weight was measured daily and individually to determine the water lost by evapotranspiration. The weight of the vessels containing control plants was measured, and the soil received water to field capacity, with no water added in the remaining treatment. The daily evapotranspiration average measured before the onset of experiments 1 and 2 was considered 100%. The gathered data was then calculated from this initial value, as follows: evapotranspiration (%) = day evapotranspiration (mL) × 100 (%)/mean evapotranspiration before stress (mL). After submitting the data for normality analyses using the Shapiro–Wilk test, an statistical analysis was performed by the Student’s *t*-test at 5% probability using GraphPad.

The parameters of leaf gas exchange [net CO_2_ assimilation rate (*A*), transpiration rate (*E*), stomatal conductance to water vapor (*gs*), and intercellular CO_2_ concentration (*Ci*)] were measured using a portable infrared gas analyzer LI-COR Mod. 6400XT (LI-COR, Lincoln, NE, United States) equipped with a measuring chamber (2 × 3 cm) with artificial light system LI-COR Mod. 6400-02B. We used the OPEN software version 6.3 to extract the data. The block temperature was 25°C, PAR was 1,500 μmol/m^2^/s, the relative humidity of the air inside the measuring chamber was between 50 and 60%, the airflow index was 400 μmol/s, and the CO_2_ concentration was 400 ppm in the reference cell, using the model 6400-01 CO2 mixer with cylinder CO_2_ (7.5 g). After submitting the gas exchange data to the Kruskal-Wallis test, we applied the Dunn’s test (*p* < 0.05) to those data with significant differences between treatments. The gas exchange measurements were on the middle third of the apical leaf, in a previously marked area, between 9:00 and 11:00 a.m., only in the first experiment.

### Transcriptomics analysis

Apical leaves from three control and three drought-stressed plants, collected 14 days after setting up the treatments (DAT), were immediately immersed in liquid nitrogen and stored at −80°C until RNA extraction, library preparation, and sequencing. Total RNA isolation, as well as the RNA quantity and quality analysis, were performed as described in Salgado et al. (2021). The GenOne Company (Rio de Janeiro, RJ, Brazil) performed the RNA-Seq using an Illumina HiSeq platform and the paired-end strategy. The Functional Genomics Center / ESALQ-USP (Piracicaba, SP, Brazil) performed the small RNAs sequencing using an Illumina HiSeq platform.

The OmicsBox version 1.3 (OmicsBox, 2019) was employed to perform all RNA-Seq analyses, using the same pipeline of analysis described previously in Salgado et al. (2021). Here, we also used FastQC (Andrews, 2018) and Trimmomatic (Bolger et al., 2014) for quality control, STAR (Dobin et al., 2013) to align the high-quality reads to the oil palm genome (Singh et al., 2013), and HTseq to quantify expression at the gene or transcript level (Anders et al., 2015). The small RNA raw data was also submitted to the same pipeline of analysis described previously in Salgado et al. (2021); and here we generated adapter-free small RNA reads using the Cutadapt software (Martin, 2011), mapped them to the reference genome using Bowtie2 (Langmead et al., 2009). The following parameters were used to run bowtie: -a (report all alignments per read) and -V 0 (no mismatches were allowed). The oil palm genome (Singh et al., 2013) – files downloaded from NCBI (BioProject PRJNA192219; BioSample SAMN02981535) on October 2020 – was again used as reference genome.

All adapter-free small RNA sequences (stressed and control) were concatenated into a single file for miRNA prediction. The miRNA prediction was made using mireap version 0.2^2^ and Shortstack version 3.4,^3^ independently or in an association. Both programs generate clusters of sequences lined up in genomic regions. Ideally, these clusters indicate the genomic location and the miRNA precursor, mature miRNA, and miRNA* sequences. StrucVis^4^ was used in sequences classified as Y or N15 by ShortStack and/or ShortStack-mireap for structural evaluation of miRNA. At last, manual curation was made of all miRNAs classified as Y (confirmed miRNA) or N1-N15, where N15 means that the candidate has all the correct metrics, but the miRNA* is absent (Axtell and Meyers, 2018). The length of the strings, the predicted structure of the hairpin, and the annotation by homology were evaluated in miRBase using the default criteria.^5^

The prediction of miRNA-putative target genes was performed using the psRNA-Target online program, version 2,^6^ with the same parameters used in Salgado et al. (2021). The NOISeq R package (Tarazona et al., 2015) was used for the analysis of differential expression of miRNAs, having the individual counts of each sample as input. The genes that showed *p*-values ≥ 0.95 were designated as differentially expressed (DE).

### Determination of biomass and soil water potential

Shoots and roots from three control and three drought-stressed plants, collected 14 days after setting up the treatments (DAP), were taken apart and weighed for fresh biomass determination and then dried in a forced-air oven at 65°C to constant weight to determine dry biomass. The leaf relative water content (RWC) was measured after 14 DAP in the oil palm plants; as well as the water potential (Ψw) of the substrate.

After harvesting the plants and homogenizing the soil, three samples were collected per pot to analyze soil water potential, using the WP4C equipment (Dew Point PotentiaMeter, METER Group, Inc.). Soil samples were placed in the equipment in the accurate reading mode, as indicated by the manufacturer. The results allow the comparison between the different treatments concerning the energy state of the soil water, which refers to the soil water content. After submitting the data for normality analyses using the Shapiro–Wilk test, an statistical analysis was performed by Student’s *t*-test at 5% probability, using GraphPad.

### Correlation analysis of differentially expressed miRNAs and mRNAs under two distinct scenarios – salinity and drought stresses

To perform correlation analysis of differentially expressed (DE) miRNAs and their respective putative target genes under two distinct scenarios, we used sets of data having the DE miRNAs and mRNAs from this present study and re-used the respective ones from Salgado et al. (2021). First, to check the data distribution, we used the Data Overview module of the Omics Fusion (Brink et al., 2016), the web platform for integrative analysis of Omics data,^7^ and then the Scatter Plot one for the correlation analysis between the sets of data—a pairwise combination of the different molecules and scenarios evaluated. The input data used was the Log_2_ (FC) data of the DE miRNAs and the DE target genes obtained from the single-omics analysis.

## Results

### Morphophysiological responses of young oil palm plants to drought stress

**Figure 1:**
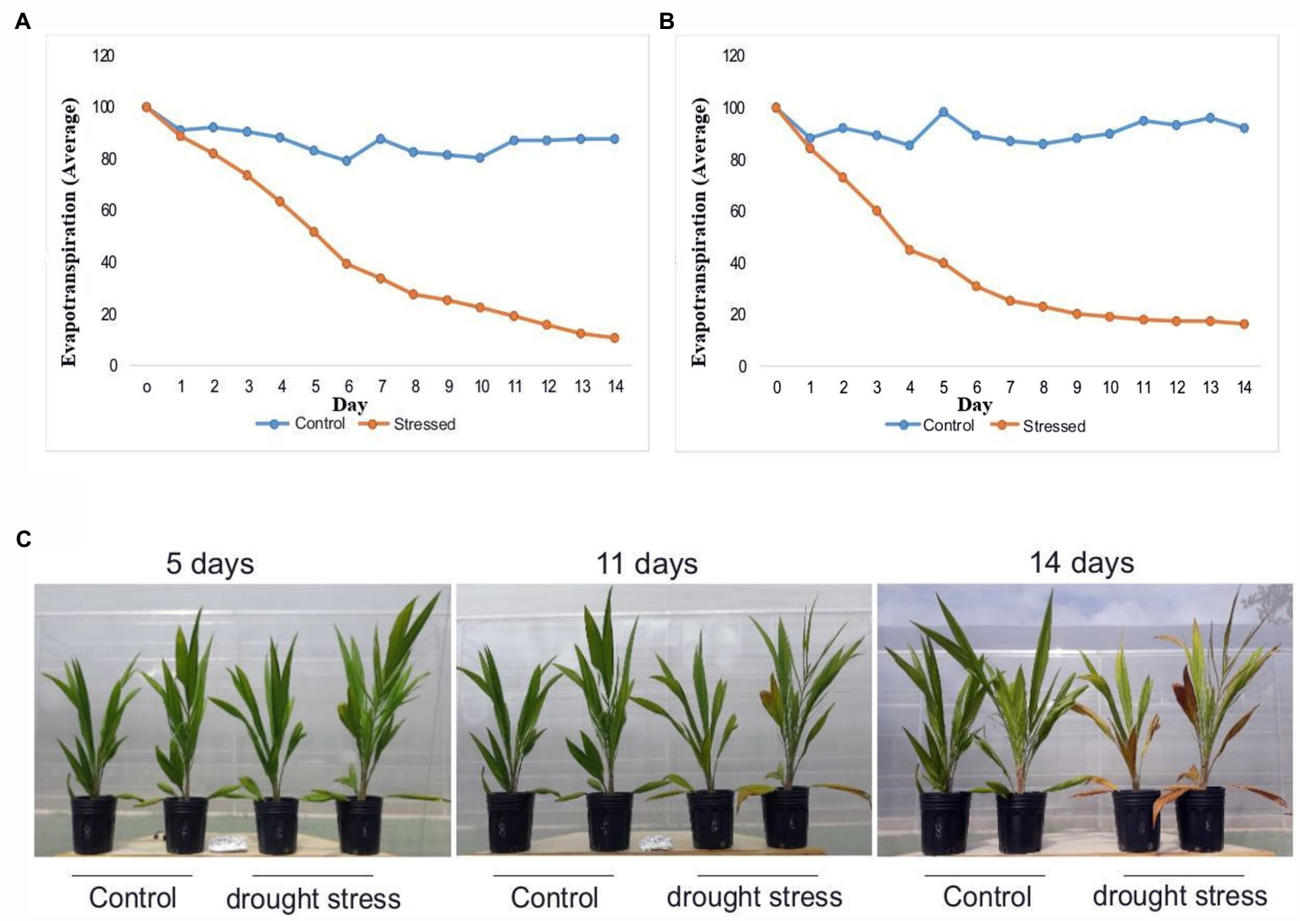
Morphophysiological responses of young oil palm plants to drought stress: **(A)** average daily rate of evapotranspiration of irrigated and non-irrigated oil palm plants from experiment 1, **(B)** average daily rate of evapotranspiration of irrigated and non-irrigated oil palm plants from experiment 2, **(C)** morphological changes of oil palm at 5, 11, and 14 days of drought stress. Evapotranspiration (Average): Percentage of the initial value measured in day 0.

The evapotranspiration rate of the control plants remained high and constant during experiments 1 and 2. Meanwhile, the water deficit caused a gradual reduction in the evapotranspiration rates in the drought-stressed plants, in both experiments (Figures 1A,B). Oil palm plants kept for 14 days under drought stress started to show morphological changes in the leaves, such as yellowing and necrosis of the edge and tip (Figure 1C). The leaf relative water content (RWC) in the oil palm plants subjected to drought stress for 14 days dropped ~50% compared to the control plants. The water potential (Ψw) of the substrate at the end of the experiment averaged 0.19 MPa in the control treatment and −13.60 in the drought-stressed one.

The stressed plants showed a reduction in shoots and roots fresh weight, averaging 87.19 and 61.59 g, respectively. Meanwhile, the control plants averaged 122.18 and 220.34 g, respectively. Such a significant reduction did not happen in either shoot or root dry weight, where stressed plants averaged 36.37 and 40.23 g, compared to an average of 36.85 and 55.44 g, respectively, for the control plants.

**Figure 2:**
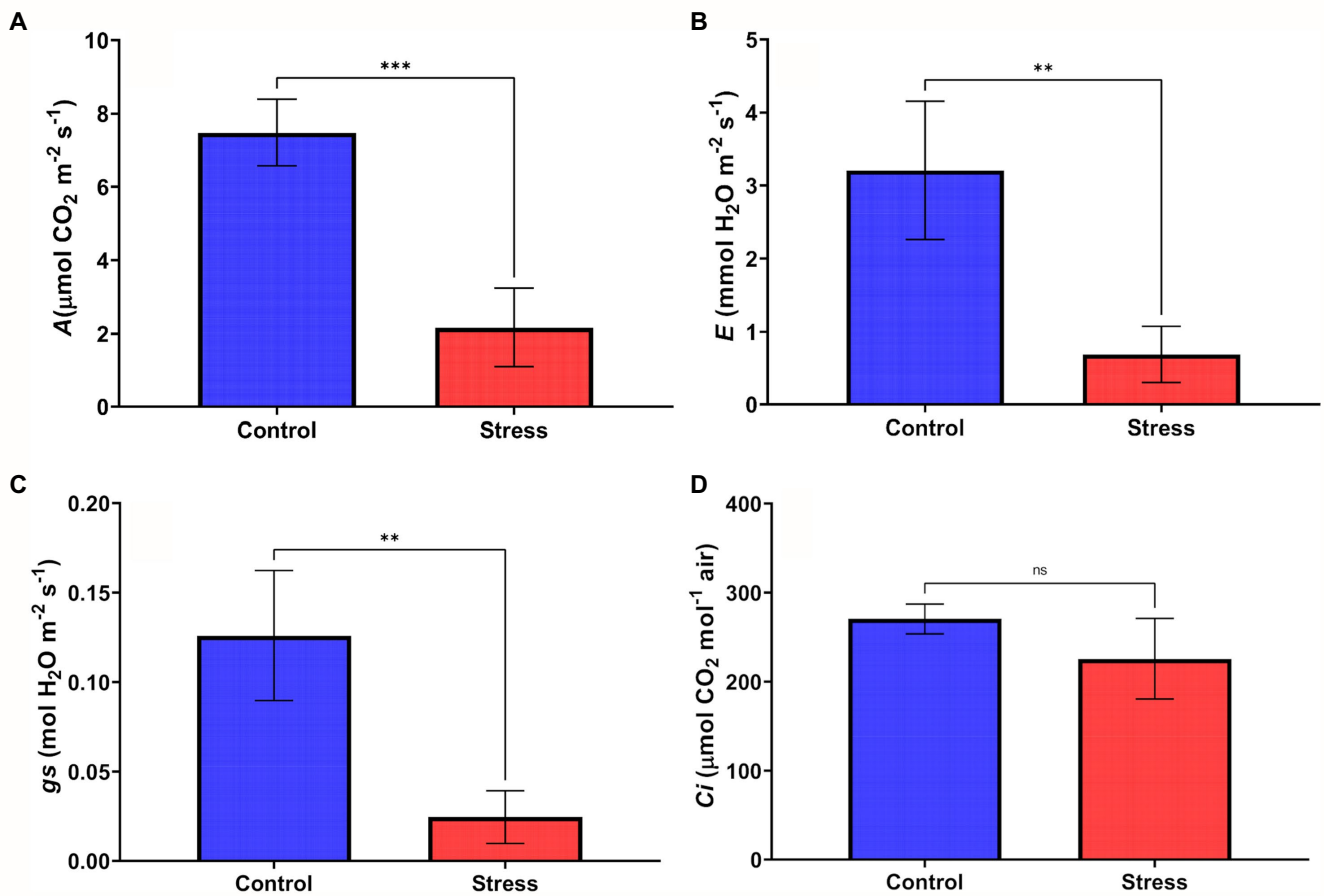
Gas exchange rates in leaves of young oil palm plants subjected to 14 days of drought stress in greenhouse conditions: **(A)** net CO_2_ assimilation rate – *A*; **(B)** transpiration rate – *E*; **(C)** stomatal conductance – *gs*; and **(D)** intercellular CO_2_ concentration – *Ci*. After submitting the gas exchange data to the Kruskal-Wallis test, Dunn’s test (*p* < 0.05) was applied to data with significant differences between treatments. ns - not significantly different, and ** or *** - significantly different.

The reduction in the Ψw led to a reduction in most of the gas exchange parameters assessed. The net CO_2_ assimilation rate (*A*), the stomatal conductance rates (*gs*), and the transpiration rate (*E*), showed a significant reduction of 81.03%, 87.74%, and 86.17%, respectively, in comparison with their respective control plants (Figures 2A–C). The intracellular concentration of CO_2_ (Ci) in the drought stressed plants showed a low percentage of reduction (19.28%) when compared to the control, with no statistical difference (Figure 2D).

### Identification of known and novel miRNAs and differential expression analysis of miRNAs

The raw reads generated ranged from 14.2 and 57.6 million per sample, presenting an extensive resource for discovering miRNAs (Table 1). After filtering out low-quality reads and removing adapters, the number of remaining clean reads ranged from three and 15 million. A total of 85,871,550 sequences, ranging from 20 to 24 nts in length, was then submitted to further analysis in the Rfam version 12.0 database, in order to remove non-coding RNAs – rRNA, tRNA, snRNA and snoRNA. The miRNA prediction was performed after mapping the remaining 61,666,402 small RNA sequences (Table 1) against the oil palm reference genome and concatenating them into a single file, generating 5,701 positive hits, from which 163 were Y and 5,538 were N15. The miRNA prediction was performed after mapping the remaining 61,666,402 small RNA sequences (Table 1) against the oil palm reference genome and concatenating them into a single file, generating 5,701 positive hits, from which 163 present all necessary characteristics, including the exact miRNA-star, to be annotate as a miRNA (code Y), and 5,538 were classified as a “maybe” (N15), in accordance with miRNA analysis codes from Shortstack, requiring a manual curation.

**Table 1:** Oil palm sRNA statistics and clean read length distribution.

**Samples**	**Raw reads (%)**	**After Cutadapt (20–24 nt)**	**After Rfam (20–24 nt)**
Control_R1_Lane1	17,359,260 (100%)	5,701,540 (32.84%)	4,405,869 (25.38%)
Control_R1_Lane2	19,137,482 (100%)	6,283,384 (32.83%)	4,809,227 (25.13%)
Control_R2_Lane1	19,766,560 (100%)	6,304,031 (31.89%)	5,179,368 (26.20%)
Control_R2_Lane2	21,788,523 (100%)	6,965,496 (31.97%)	5,682,583 (26.08%)
Control_R3_Lane1	25,695,279 (100%)	5,769,021 (22.45%)	3,307,121 (12.87%)
Control_R3_Lane2	28,288,139 (100%)	6,370,094 (22.52%)	3,560,220 (12.59%)
Drought_R1_Lane1	23,959,300 (100%)	6,471,468 (27.01%)	4,410,743 (18.41%)
Drought_R1_Lane2	26,514,939 (100%)	7,182,696 (27.09%)	4,824,005 (18.19%)
Drought_R2_Lane1	14,201,378 (100%)	3,063,342 (21.57%)	1,825,359 (12.85%)
Drought_R2_Lane2	15,714,905 (100%)	3,063,342 (19.49%)	1,978,270 (12.59%)
Drought_R3_Lane1	52,289,340 (100%)	13,652,218 (26.11%)	10,366,551 (19.83%)
Drought_R3_Lane2	57,646,162 (100%)	15,044,918 (26.10%)	11,317,086 (19.63%)

A total of 81 miRNAs resulted from a manual curatorship evaluating the length of the strings (20–22 nts), predicting the structure of the hairpin by strucVis version 0.4,^8^ and annotation by homology in the miRBase database,^9^ being 52 conserved miRNAs already reported in other species and 29 oil palm-specific miRNAs (Table 2). It is a fact that plant miRNAs are generally 20 to 22 nt in size, with 23 and 24 being rare (Axtell and Meyers, 2018). The 24 nt sequences represent siRNAs, and none of them was pointed out as a possible miRNA by the Shortstack and mireap programs after the curation process employed in this study, demonstrating the robustness of the prediction, annotation, and curation process used. Among the 29 new miRNAs, 27 were similar to those already reported by Salgado et al. (2021) in oil palm under salinity stress – egu-miR01sds to egu-miR27sds. The two new miRNAs identified in this study, egu-miR28ds, and egu-miR29ds, have a length of 21 nts and are located in intragenic regions of the *E. guineensis* genome (Table 2). The former is the target GTPase-activating protein GYP7 gene (LOC105043478) and has a length of 144 nts, while the latter is in an uncharacterized protein (LOC105044755) and has 101 nts. The GTPase-activating protein GYP7 gene is 9,498 nts long, has five exons, and code for two protein isoforms in the *E. guineensis* genome. The egu-miR28ds gene is located in the third and longest exon. The uncharacterized LOC105044755, on the other hand, is 2,165 nts long and has no intron, and the egu-miR29ds gene is located pretty much in the middle of it.

**Table 2:** The two new oil palm-specific miRNAs predicted in this study, and their putative target genes predicted using psRNA-Target online program, version 2.

**miRNA**	**miRNA size**	**Mireap**	**Shortstack**	**miRNA gene size**
egu-miR28ds	21 nt	o	–	144 nt
**Location**	Gene ID*	**Gene description**	**miRNA_Aligned Fragment**	
intragenic	LOC105043478	GTPase-activating protein GYP7	UAGUAGUCUCCAAAUCACAUG	
**Alignment**	**Target Gene_Aligned Fragment**	**Target Gene ID**	**Target Gene Description**	
.:::::::::::::::.::.	5′ UAUGUGAUUUGAAGACUGCUG 3′	LOC105039667	Probable protein-S-isoprenylcysteine O-methyltransferase	
.::::::::::.::::::	5′ UGUGAAAUUUGGAGGCUACUA 3′	LOC105050641	GBF-interacting protein 1-like	
.::::::::::.::::::	5′ UGUGAAAUUUGGAGGCUACUA 3′			
.::::::::::.::::::	5′ UGUGAAAUUUGGAGGCUACUA 3′			
.::::::::::.::::::	5′ UGUGAAAUUUGGAGGCUACUA 3′			
**miRNA**	**miRNA size**	**Mireap**	**Shortstack**	**miRNA gene size**
egu-miR29ds	21 nt	o	o	101 nt
**Location**	Gene ID*	**Gene description**	**miRNA_Aligned Fragment**	
intragenic	LOC105044755	Uncharacterized – protein coding	UCUCGGGCGGCGACCUCCUCC	
**Alignment**	**Target Gene_Aligned Fragment**	**Target Gene ID**	**Target Gene Description**	
::::::::::::::::::::	5′GGAGGAAGUCGCCGCCCGAGA 3′	LOC105054028	Probable xyloglucan galactosyltransferase GT19	
::::::::::::::::::	5′GGAAGAGGUCGCCGCCGGAGC 3′	LOC109506582	Probable protein phosphatase 2C 8	
::::::::::::::::::	5′UGAGGCGG-CGCCGCCCGAGA 3′	LOC105041954	Protein FATTY ACID EXPORT 2	
::::::::::::::::::	5′UGAGGCGG-CGCCGCCCGAGA 3′	LOC105053017	Protein FATTY ACID EXPORT 7	
:::::::.::::::::::.	5′GGAUGCGGUUGCCGCCCGAGG 3′	LOC105041155	EF1A lysine methyltransferase 2	

**Figure 3:**
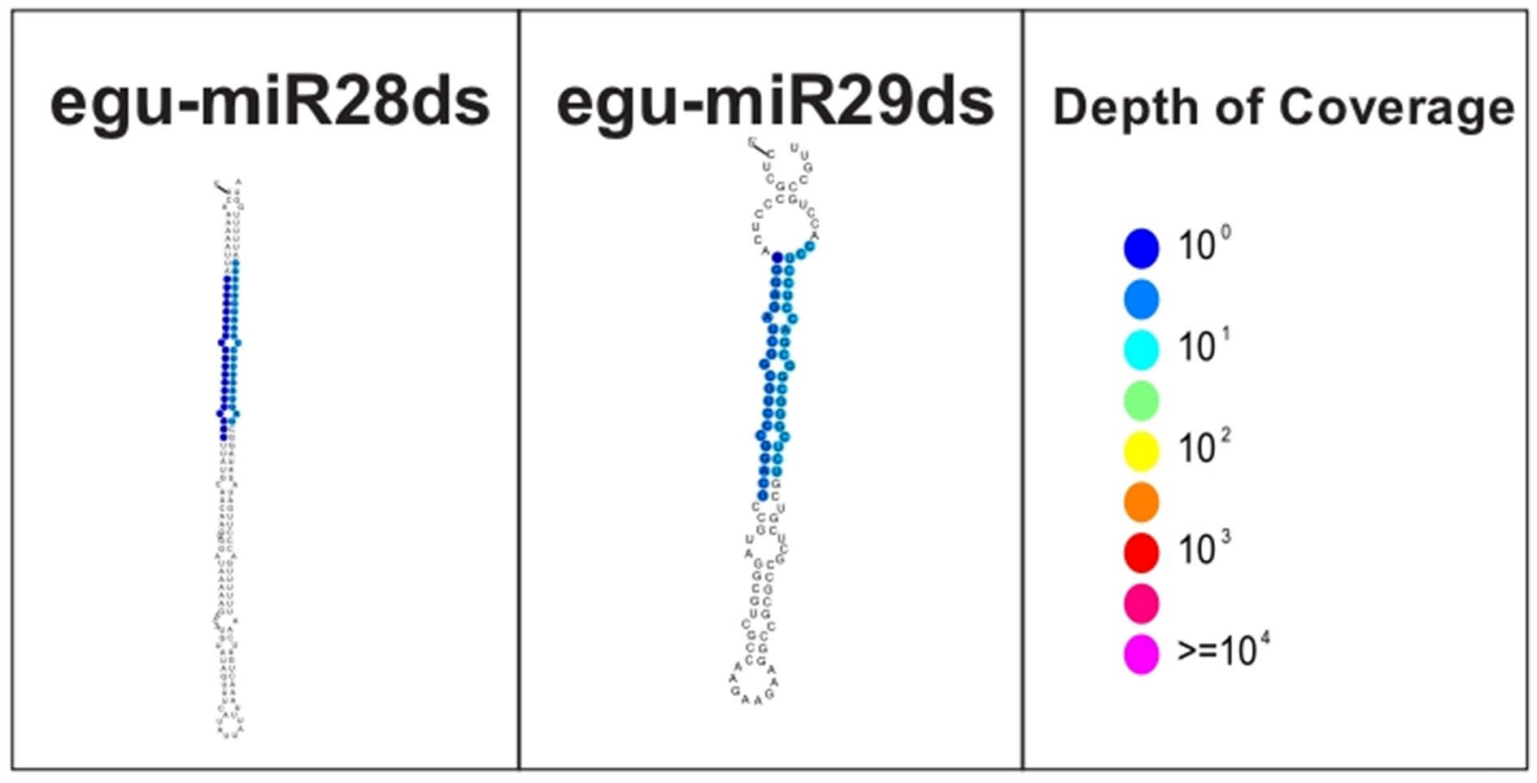
Structure of the new miRNAs identified in oil palm under water deficit.

To avoid the annotation of false-positive miRNAs, we followed the curation criteria suggested by Axtell and Meyers (2018), evaluating the predicted structure of the hairpin of the new miRNAs (Figure 3), both show precise excision of the initial stalk duplex of a precursor RNA, without secondary stalks or large internal loops (greater than five nts), with a processing precision above 94%.

Sixty-two out of the 81 known and novel miRNAs showed a significant (probability ≥0.95) different level of expression under drought stress. While miR396e, miR159b, miR529b, egu-miR19sds, and egu-miR29ds were up-regulated, the remaining 57 miRNAs had their expression level reduced, compared to the level in the control treatment (Supplementary Table 1). The ppe-miR397 miRNA appeared in different positions of the genome, with distinct differential expression levels, and, at position NC_025997.1:28868274-28868388, it was down-regulated (*p* < 0.97), while at position NC_025993.1:61023997-61024130 it did not show significant (*p* < 0.77) differential expression (Supplementary Table 1).

### Prediction and differential expression analysis of miRNAs-putative target genes

The psRNA-Target online program – version 2^10^ – led to 357 positive hits as DE miRNA-putative target genes. Based on the LOC Ids from the oil palm reference genome (Singh et al., 2013), there were 185 distinct putative target genes out of the 357 positive hits. All 62 DE miRNA had two or more putative target genes.

Almost 100% of the high-quality read pairs in all samples mapped to the oil palm reference genome, which had 29,567 genomic features of type “gene” retrieved from 2,781 ref. sequences in GCF_000442705.1_EG5_genomic.fna file; however, 4,115 of these features had no aligned reads detected in any of the samples (Table 3).

**Table 3:** Statistics of RNA-Seq data from six samples of oil palm plants subjected to two treatments (control and drought stress); three replicates per treatment.

Sample*	Control treatment			Stressed treatment		
	R1	R2	R3	R1	R2	R3
Input high quality read pairs	28,506,037	27,758,299	27,482,363	29,085,207	29,056,879	30,875,128
Aligned reads to feature	15,462,284	23,380,536	14,294,486	17,136,348	20,031,805	25,942,349
Aligned reads to no feature	4,422,784	2,283,704	4,201,816	5,803,528	4,325,704	2,977,275
Ambiguous	230.22	401.78	225.45	199.44	228.05	315.77
Alignment not unique	8,390,751	1,692,282	8,760,612	5,945,893	4,471,325	1,639,731
Low alignment quality	0	0	0	0	0	0
Not aligned	2	2	0	0	0	0

Mapping to reference genome EG5 (BioProject PRJNA192219 and BioSample SAMN02981535) available at NCBI.

^*^29,567 genomic features of type “gene”, retrieved from 2,781 ref sequences in GCF_000442705.1_EG5_genomic.fna & 4,115 features (13.92%) for with no aligned reads was detected in any of the samples.

When comparing drought-stressed against control plants, the pairwise differential expression analysis revealed that out of the 185 distinct miRNA-putative target genes, 88 differentially expressed at False Discovery Rate (FDR) ≤ 0.05; being 44 positively regulated (Log_2_(FC) > 0) and 44 negatively regulated (Log_2_(FC) < 0). The down-regulated miRNA-putative target genes experienced a reduction in the expression level, ranging from 28% to almost 100%. On the other hand, the up-regulated miRNA-putative target genes increased from 1.28X to 166.24X (Table 4).

**Table 4:** Profile of differential expressed miRNA and their differential expressed putative target genes from oil palm plants.

miRNA					Putative target gene				
Name	EP	Probability	FC	Log_2_FC	ID	EP	FC	Log_2_FC	FDR
ama-miR156	Down	1.00	0.54	−0.89	LOC105053992	Up	166.24	7.38	0.00
ata-miR156e-5p	Down	1.00	0.57	−0.81	LOC105053992	Up	166.24	7.38	0.00
bra-miR168a-5p	Down	1.00	0.25	−2.02	LOC105047586	Up	44.07	5.46	0.00
bra-miR168c-5p	Down	1.00	0.20	−2.33	LOC105047586	Up	44.07	5.46	0.00
ppe-miR397	Down	0.97	0.38	−1.40	LOC105046309	Up	30.64	4.94	0.00
egu-miR02sds	Down	1.00	0.01	−7.42	LOC105042156	Up	28.34	4.82	0.01
ppe-miR397	Down	0.97	0.38	−1.40	LOC105036954	Up	22.40	4.49	0.00
ata-miR528-5p	Down	0.99	0.24	−2.05	LOC105036904	Up	19.12	4.26	0.00
egu-miR20sds	Down	0.97	0.07	−3.91	LOC105046327	Up	14.61	3.87	0.01
ssp-miR827	Down	1.00	0.12	−3.04	LOC105043377	Up	12.69	3.67	0.00
ata-miR167d-5p	Down	1.00	0.31	−1.67	LOC105053405	Up	8.21	3.04	0.00
gma-miR482a-3p	Down	0.99	0.77	−0.38	LOC105043410	Up	7.96	2.99	0.00
egu-miR25sds	Down	0.97	0.23	−2.10	LOC105036224	Up	7.73	2.95	0.00
ata-miR166d-3p	Down	1.00	0.15	−2.78	LOC105046708	Up	6.30	2.66	0.00
atr-miR166b	Down	1.00	0.27	−1.88	LOC105046708	Up	6.30	2.66	0.00
osa-miR166i-3p	Down	1.00	0.13	−2.99	LOC105046708	Up	6.30	2.66	0.00
sly-miR166c-3p	Down	0.99	0.32	−1.63	LOC105046708	Up	6.30	2.66	0.00
ata-miR396e-5p	Down	0.97	0.49	−1.03	LOC105033560	Up	4.72	2.24	0.03
ata-miR160c-5p	Down	1.00	0.13	−2.99	LOC105044300	Up	4.66	2.22	0.00
egu-miR07sds	Down	1.00	0.23	−2.09	LOC105044919	Up	4.34	2.12	0.00
ata-miR160c-5p	Down	1.00	0.13	−2.99	LOC105038384	Up	3.49	1.80	0.00
aof-miR395a	Down	1.00	0.09	−3.45	LOC105041393	Up	3.45	1.79	0.00
atr-miR535	Down	0.99	0.57	−0.82	LOC105051200	Up	3.44	1.78	0.00
mtr-miR2673b	Down	0.99	0.03	−5.21	LOC105060921	Up	3.44	1.78	0.00
mdm-miR171b	Down	1.00	0.34	−1.57	LOC105043623	Up	3.12	1.64	0.00
osa-miR2118p	Down	1.00	0.58	−0.78	LOC105054413	Up	3.05	1.61	0.00
egu-miR04sds	Down	1.00	0.00	−7.79	LOC105040907	Up	2.86	1.51	0.00
bra-miR319-3p	Down	1.00	0.27	−1.89	LOC105032078	Up	2.71	1.44	0.00
atr-miR156c	Down	1.00	0.55	−0.87	LOC105061255	Up	2.67	1.42	0.03
bdi-miR529-5p	Down	1.00	0.24	−2.08	LOC105061255	Up	2.67	1.42	0.03
egu-miR07sds	Down	1.00	0.23	−2.09	LOC105061572	Up	2.59	1.37	0.00
osa-miR2118p	Down	1.00	0.58	−0.78	LOC105054674	Up	2.50	1.32	0.00
egu-miR18sds	Down	0.97	0.32	−1.63	LOC105055689	Up	2.45	1.29	0.00
egu-miR15sds	Down	0.99	0.32	−1.64	LOC105044088	Up	2.41	1.27	0.00
egu-miR05sds	Down	1.00	0.12	−3.09	LOC105045735	Up	2.40	1.26	0.00
egu-miR10sds	Down	1.00	0.15	−2.70	LOC105043130	Up	1.78	0.83	0.00
ata-miR528-5p	Down	0.99	0.24	−2.05	LOC105055547	Up	1.77	0.82	0.00
aof-miR395a	Down	1.00	0.09	−3.45	LOC105038678	Up	1.67	0.74	0.00
aof-miR391	Down	0.99	0.09	−3.53	LOC105045520	Up	1.63	0.71	0.00
egu-miR11sds	Down	0.99	0.08	−3.62	LOC105050858	Up	1.63	0.70	0.00
egu-miR10sds	Down	1.00	0.15	−2.70	LOC105034850	Up	1.62	0.69	0.00
ssp-miR827	Down	1.00	0.12	−3.04	LOC105034273	Up	1.60	0.68	0.00
egu-miR02sds	Down	1.00	0.01	−7.42	LOC105047587	Up	1.60	0.68	0.01
ata-miR169i-3p	Down	1.00	0.07	−3.92	LOC105043671	Up	1.60	0.68	0.00
ata-miR528-5p	Down	0.99	0.24	−2.05	LOC105034839	Up	1.52	0.61	0.00
ata-miR169i-3p	Down	1.00	0.07	−3.92	LOC105060868	Up	1.47	0.55	0.00
egu-miR08sds	Down	1.00	0.11	−3.22	LOC105042656	Up	1.45	0.53	0.00
bra-miR168a-5p	Down	1.00	0.25	−2.02	LOC105051551	Up	1.39	0.48	0.01
bra-miR168a-5p	Down	1.00	0.25	−2.02	LOC105042722	Up	1.28	0.36	0.04
ssp-miR827	Down	1.00	0.12	−3.04	LOC105054157	Down	0.00	−7.75	0.00
ata-miR169i-3p	Down	1.00	0.07	−3.92	LOC105051537	Down	0.03	−5.26	0.00
ata-miR399a-3p	Down	1.00	0.01	−6.21	LOC105035561	Down	0.03	−5.13	0.00
ata-miR395c-3p	Down	1.00	0.08	−3.64	LOC105048606	Down	0.04	−4.60	0.00
egu-miR20sds	Down	0.97	0.07	−3.91	LOC105032247	Down	0.06	−4.18	0.00
egu-miR08sds	Down	1.00	0.11	−3.22	LOC105048722	Down	0.14	−2.82	0.00
atr-miR159	Down	1.00	0.23	−2.13	LOC105043705	Down	0.16	−2.65	0.00
egu-miR05sds	Down	1.00	0.12	−3.09	LOC105041381	Down	0.16	−2.61	0.00
egu-miR03sds	Down	0.99	0.48	−1.05	LOC105059001	Down	0.17	−2.58	0.00
aof-miR391	Down	0.99	0.09	−3.53	LOC105039220	Down	0.17	−2.57	0.00
mes-miR399f	Down	1.00	0.01	−6.62	LOC105052027	Down	0.20	−2.29	0.00
egu-miR09sds	Down	1.00	0.21	−2.22	LOC105041147	Down	0.22	−2.21	0.00
ata-miR396e-5p	Down	0.97	0.49	−1.03	LOC105033558	Down	0.25	−2.02	0.00
ata-miR528-5p	Down	0.99	0.24	−2.05	LOC105048962	Down	0.29	−1.77	0.00
ata-miR169i-3p	Down	1.00	0.07	−3.92	LOC105047549	Down	0.31	−1.71	0.03
ata-miR390-5p	Down	0.98	0.22	−2.20	LOC105053593	Down	0.31	−1.70	0.00
bdi-miR169c-3p	Down	1.00	0.08	−3.67	LOC105046060	Down	0.33	−1.61	0.03
gma-miR482a-3p	Down	0.99	0.77	−0.38	LOC105058639	Down	0.33	−1.58	0.00
egu-miR01sds	Down	1.00	0.16	−2.60	LOC105055591	Down	0.34	−1.56	0.00
egu-miR04sds	Down	1.00	0.00	−7.79	LOC105052116	Down	0.36	−1.47	0.00
ata-miR167d-5p	Down	1.00	0.31	−1.67	LOC105049211	Down	0.38	−1.41	0.00
aof-miR395a	Down	1.00	0.09	−3.45	LOC105042288	Down	0.38	−1.41	0.00
vvi-miR828a	Down	0.96	0.58	−0.78	LOC105051514	Down	0.41	−1.28	0.00
ata-miR396e-5p	Down	0.97	0.49	−1.03	LOC105052992	Down	0.44	−1.20	0.00
ata-miR396e-5p	Down	0.97	0.49	−1.03	LOC105042167	Down	0.48	−1.05	0.00
egu-miR11sds	Down	0.99	0.08	−3.62	LOC105056373	Down	0.49	−1.02	0.00
egu-miR26sds	Down	0.97	0.13	−2.91	LOC105056373	Down	0.49	−1.02	0.00
mdm-miR171b	Down	1.00	0.34	−1.57	LOC105059511	Down	0.49	−1.02	0.00
egu-miR18sds	Down	0.97	0.32	−1.63	LOC105031985	Down	0.50	−1.01	0.00
bdi-miR530b	Down	0.99	0.21	−2.23	LOC105055997	Down	0.50	−1.00	0.00
mdm-miR171b	Down	1.00	0.34	−1.57	LOC105054013	Down	0.53	−0.92	0.00
mdm-miR171b	Down	1.00	0.34	−1.57	LOC105054987	Down	0.58	−0.79	0.00
egu-miR15sds	Down	0.99	0.32	−1.64	LOC105050166	Down	0.58	−0.78	0.01
vvi-miR828a	Down	0.96	0.58	−0.78	LOC105043777	Down	0.59	−0.77	0.00
vvi-miR828a	Down	0.96	0.58	−0.78	LOC105039801	Down	0.61	−0.70	0.00
egu-miR11sds	Down	0.99	0.08	−3.62	LOC105049000	Down	0.62	−0.69	0.01
egu-miR03sds	Down	0.99	0.48	−1.05	LOC105048718	Down	0.63	−0.66	0.00
egu-miR08sds	Down	1.00	0.11	−3.22	LOC105049925	Down	0.63	−0.66	0.00
egu-miR03sds	Down	0.99	0.48	−1.05	LOC105060476	Down	0.67	−0.58	0.00
egu-miR07sds	Down	1.00	0.23	−2.09	LOC105058953	Down	0.68	−0.56	0.02
egu-miR22sds	Down	0.99	0.06	−4.18	LOC105060538	Down	0.68	−0.56	0.02
ata-miR399a-3p	Down	1.00	0.01	−6.21	LOC105047412	Down	0.68	−0.56	0.00
mes-miR399f	Down	1.00	0.01	−6.62	LOC105047412	Down	0.68	−0.56	0.00
aof-miR395a	Down	1.00	0.09	−3.45	LOC105041383	Down	0.71	−0.50	0.00
osa-miR2118p	Down	1.00	0.58	−0.78	LOC105044139	Down	0.72	−0.48	0.02
cme-miR396e	Up	0.97	2.16	1.11	LOC105033558	Down	0.25	−2.02	0.00
egu-miR29ds	Up	0.97	4.61	2.20	LOC105053017	Down	0.71	−0.49	0.01
cme-miR396e	Up	0.97	2.16	1.11	LOC105033560	Up	4.72	2.24	0.03
egu-miR19sds	Up	0.95	1.45	0.54	LOC105049219	Up	3.62	1.86	0.00
cpa-miR159b	Up	0.97	2.32	1.21	LOC105032078	Up	2.71	1.44	0.00
osa-miR529b	Up	0.98	3.28	1.71	LOC105061255	Up	2.67	1.42	0.03

False discovery rate (FDR), Identity in the reference genome EG5 at NCBI (ID), and fold change (FC). EP, Expression profile; FC, Fold Change (Drought/Control).

### Integrating the expression profiles from DE miRNA-putative target genes and their respective DE miRNAs

The interaction between DE miRNAs and their putative target genes was investigated using Cytoscape – version 3.8.2,^11^ which led to the identification of a total of 102 miRNA-mRNA interactions, involving 62 DE miRNAs and 88 DE mRNAs. The analysis showed that a single miRNA can regulate multiple mRNAs and that a single mRNA can correlate with more than one miRNA, suggesting that the miRNA-mRNA interaction network involved in water stress is highly complex.

By integrating the expression profiles of the miRNA-putative target genes and their related miRNAs, 26 DE miRNA had just one DE putative target gene, 15 had two, eight had three, five had four, and eight had none. On the other hand, among the 88 DE putative target genes, 79 of them had just one DE miRNA, seven had two, one had three, and one had four (Table 4).

**Figure 4:**
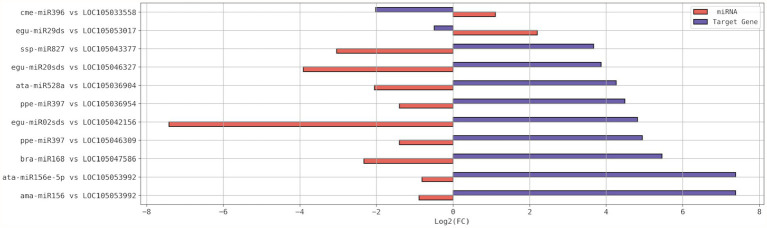
Expression profiles in Log_2_(FC) of the differentially expressed (DE) miRNAs and their respective DE putative target gene(s), resulted from submission of young oil palm plants to drought stress. FC, Fold Change.

miR396e and egu-miR29ds, with a respective increment of 116% and 361%, were up-regulated due to drought stress, while their putative target genes had their expression level reduced to 25% and 71%, respectively (Table 4; Figure 4). LOC105053992 was the putative target gene that experienced the highest increase in expression, 166.24X higher than in the control plant. It experienced regulation by two miRNAs (ama-miR156 and ata-miR156e-5p) that were down-regulated to about 50% of the level in the control plant (Figure 4).

### Functional annotation of the differentially expressed target genes

The target genes underwent functional annotation using the InterProScan database. Among the 88 DE target genes selected for functional annotation, 32 had positive hits for biological processes, 50 for molecular function, and 19 for cellular components. Transmembrane transport (GO:0055085) and regulation of transcription by DNA (GO:0006355) had the highest number of biological processes present, five; followed by hormone response processes (GO: 0009725), protein phosphorylation (GO: 0006468), pigment biosynthetic process (GO: 0046148), histone lysine methylation (GO: 0034968), lipid metabolic process (GO: 0006629), oxidation–reduction process (GO: 0055114) and histone methylation (GO: 0016571), with two each (Supplementary Table 2).

For molecular functions, the GO terms that appeared the most were protein binding (GO: 0005515), with nine occurrences, followed by DNA binding (GO: 0003677), with eight, and ATP binding (GO: 0005524), with seven. The cellular component most frequently present was the nucleus (GO: 0005634), with seven occurrences, followed by the membrane (GO: 0016020), with six, and the integral membrane component (GO: 0016021), with four (Supplementary Table 2).

When analyzing the most expressed domains, TF_GRAS (IPR005202) came first with 4, followed by SPX domain (IPR004331) with three, and SET_dom (IPR001214), Tyrosinase_Cu-bd (IPR002227), Protein kinase domain (IPR000719), SANT/Myb (IPR001005), and SBP_dom (IPR004333) with two hits each. MFS family, with three members, was the largest one, followed by the ABC, Polyphenol_oxidase, and Hist-Lys_N-MeTrfase_plant families with two members each (Supplementary Table 2).

To further characterize the putative target genes, the LOC numbers were used to identify the ID of the protein coded by them. Nine LOC numbers were from genes coding for ncRNAs, and the remaining 79 led to the identification of 138 proteins (distinct XP id). These 138 proteins were submitted to analysis in the GhostKOALA platform (Kanehisa et al., 2016), and in the EggNOG v5.0 platfom (Huerta-Cepas et al., 2019; Cantalapiedra et al., 2021). The results of the hierarchical, functional and phylogenetic annotation using the EggNOG platform, having the Liliopsida class as taxonomic scope, is presented in Supplementary Table 3; only 132 out of the 138 proteins were annotated. Only 44 proteins were annotated in the GhostKOALA platform, being 11 transcription factors from three families – MYB, HOX, and NF-Y.

### Correlation analyses of DE miRNA-putative target genes and their respective DE miRNAs

**Figure 5:**
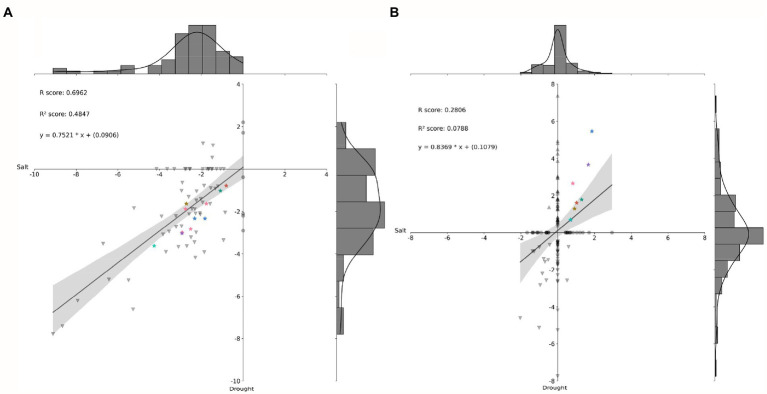
Histogram and correlation analysis of the Log_2_ (FC) of differentially expressed (DE) miRNAs **(A)** and their respective DE putative target genes **(B)**, by pairwise comparison of two scenarios: Salt stress and Drought stress. Stars of same color identify miRNAs (in A) and their respective putative target genes upregulated in both stresses (in B). FC, Fold Change.

First, 72 miRNAs differentially expressed in young oil palm plants under salinity stress (Salgado et al., 2021) were submitted to correlation analysis against 62 from the present study (Figure 5A). Then, 51 DE miRNA-putative target genes from Salgado et al. (2021) underwent correlation analysis against the 88 from the present study (Figure 5B). These results revealed eight miRNA-putative target genes upregulated in both scenarios, salinity and drought stress (Figure 5B), that also had the miRNAs targeting them down-regulating in those scenarios (Figure 5A). Among them, there were three genes expressing lncRNAs, two coding for proteins from the Major facilitator superfamily, one for a putative histone-modifying enzyme harboring a demethylase activity, one for a protein from the class III homeodomain-leucine zipper family, and one for a protein from the Macrophage migration inhibitory factor (MIF) family.

## Discussion

Palms are considered the third most important economic group of plants, second only to grasses and legumes (Meerow et al., 2012). miRNAs have been described as master regulators of gene expression and involved in controlling growth and development and plant responses to different stresses (Liu et al., 2008; Ding et al., 2009; Djami-Tchatchou et al., 2017). Still, little information on palm miRNA is available (Md Nasaruddin et al., 2007; Low et al., 2014; da Silva et al., 2016; Ho et al., 2017; Zheng et al., 2019; Salgado et al., 2021), and none of them studied the miRNAs involved in the response of oil palm plants to drought stress. Thus, this is the first report of prospecting oil palm miRNAs and analyzing their expression profile together with their target genes under water deprivation.

The reduction of *gs* and Ψw seen in the present study, inducing an abrupt stomatal closure, is a shred of evidence that oil palm has a high sensitivity to drought. It suggests that mechanisms for water control mediate the decrease in *gs*; in other words, that water deprivation is sensed by the roots, triggering the production of abscisic acid (ABA), which results in stomatal closure mediated by a transduction cascade (Chaves et al., 2009; Cutler et al., 2010; Silva et al., 2016). The effects of *A* reduction during water deficit (Figure 2A) may be related to the increase in diffusive (stomatal and mesophilic restrictions) and biochemical limitations (Chaves et al., 2009; Flexas et al., 2012).

This study identified 81 miRNAs, where 52 miRNAs are deeply conserved among various plant species, such as *A. thaliana* (Liu et al., 2008), *O. sativa* (Zhou et al., 2010), *Populus trichocarpa* (Lu et al., 2008), and *M. truncatula* (Wang et al., 2011). Some of them, miR156, miR160, miR166, miR167, miR168, miR172, miR396, miR528, and miR535, have already been found in oil palm by Ho et al. (2017) in floral meristems, while Fang et al. (2013) identified miR156, miR395, and miR528 in the mesocarp of oil palm fruits. Also, Md Nasaruddin et al. (2007) identified miR156, miR159, and miR160 in oil palm’s apical meristem and immature and mature flowers. The other 29 miRNAs are specific for oil palm (Table 3), from which 27 have been already reported in our previous study (Salgado et al., 2021) – regarding oil palm responses to salinity stress, and two (egu-miR28ds and egu-miR29ds) reported for the first time.

Sixty-two out of the 81 miRNAs showed significant differential expression. While five of them up-regulated under drought stress, the remaining 57 were down-regulated (Table 4). That is a behavior similar to the one seen usually in genes encoding proteins – meaning that a miRNA expression is also up-or down-regulated in response to stress (Fang et al., 2013), as also shown for *A. thaliana* (Liu et al., 2008), *O. sativa* (Zhou et al., 2010), *P. trichocarpa* (Lu et al., 2008), *M. truncatula* (Wang et al., 2011), among others.

A series of transcription factor (TFs) putative genes were also targeted by miRNAs in such plants when submitted to drought stress, as already seen in young oil palm plants under salinity stress (Salgado et al., 2021). TFs, such as auxin response factors (ARFs), squamosa promoter binding proteins, ethylene response transcription factors, MYB transcription factor, homeobox-leucine protein zipper HOX, transcription factor GAMYB, and nuclear transcription factor Y, which are responsible for regulating plant growth and development (Wu and Poethig, 2006; Gandikota et al., 2007), were predicted as putative miRNA-target genes in oil palm under water deficit in this study.

Most of these miRNAs targeting TFs were down-regulated (miR156, miR160c-5p, miR166d-3p, miR169i-3p, miR156c, miR166b, miR529-5p, miR319-3p, egu-miR02sds, egu-miR07sds, egu-miR11sds, egu-miR26sds, miR171b, miR166i-3p, miR166c-3p), indicating that the decrease in these miRNAs will increase the expression of some corresponding transcription factors, thus promoting the activation of a set of encoding genes to play defensive roles against abiotic stresses. This behavior is present in other species under water deficit (Liu et al., 2008; Zhou et al., 2010).

The plant response to drought stress is very similar to the initial plant response to salinity stress, and many common occurrences between these two primary stresses are also expected (Uddin et al., 2016). Salgado et al. (2021) observed that miRNAs miR166, miR169, miR319, miR396, and miR529 shows reduced expression profiles in young oil palm plants subjected to salt stress, a similar behavior to what happened in oil palm plants under drought stress. It is proved, through functional analysis, that these miRNAs regulate the levels of transcription of TFs, thus affecting the levels of TF proteins.

miR529 in the leaves of oil palm plants under salinity (Salgado et al., 2021) and drought stress showed a reduction of 80% and 76% in expression levels, respectively. Both showed up-regulation of their putative target gene – SBP (squamosa promoter-binding-like protein) – by ~70%. Squamosa promoter binding proteins are putative transcription factors with a plant-specific SBP domain consisting of 76 amino acids in length responsible for regulating various biological processes, including drought and saline stress. According to studies carried out by Hou et al. (2018), the overexpression of the homologous SBP16 gene from grapes (*Vitis vinifera*) in *A. thaliana* promoted an increase in tolerance to drought and salinity stresses during seed germination, as well as in seedlings and mature plants, regulating the signaling cascades of SOS and ROS.

egu-miR02sds showed a 99% reduction in its expression level in plants under drought stress, promoting a 60% increase in the expression of its putative target gene NF-YB (LOC105047587 – nuclear transcription factor Y subunit B-4-like), a similar behavior shown when under salt stress (Salgado et al., 2021). The Nuclear factor Y (NF-Y) is a ubiquitous transcription factor with high affinity and sequence specificity for the CCAAT box, a cis-element present in about 25% of eukaryotic gene promoters. NF-Y is a heterotrimeric complex composed of three distinct subunits (NF-YA, NF-YB, and NF-YC; Mantovani, 1999). Studies shows that NF-Y is responsible for activating a transcriptional cascade critical for drought resistance, where its overexpression improves resistance and promotes drought tolerance in arabidopsis and maize (Nelson et al., 2007; Liu et al., 2008).

According to Li et al. (2008), overexpression of NF-YA5 in arabidopsis reduced water loss in leaves and resulted in a resistance to water stress higher than the wild type. Nelson et al. (2007) showed that NF-YB overexpression promoted drought tolerance in maize, based on the responses of several stress-related parameters, including chlorophyll content, stomatal conductance, leaf temperature, reduced wilting, and maintenance of photosynthesis. Thus, NF-Y is crucial for expressing a series of genes responsive to drought stress, and its induction takes place at both transcriptional and post-transcriptional levels (Li et al., 2008; Liu et al., 2008).

The miRNAs miR168, miR395, miR396, miR397 were also down-regulated and are directly involved in the stress response process or stress tolerance (Sunkar et al., 2005; Gray-Mitsumune and Matton, 2006). miR168 regulates the *Argonaute 1* (AGO1) gene in *A. thaliana* and *P. trichocarpa* (Lu et al., 2005). AGO1 proteins can be directly associated with siRNAs / miRNAs before and after recognizing their mRNA targets and are required for normal plant development (Vaucheret et al., 2004). In the present study, miR168 expression level experienced a 75% reduction in drought-stressed plants, while its putative target gene (LOC105042722 – protein argonaute 1A-like) up-regulated 28%.

The present study led us to identify two new oil palm-specific miRNAs and enabled a comparative analysis of expression levels with miRNAs in oil palm reported under salt stress (Salgado et al., 2021). Comparative studies of miRNAs can help to increase our understanding, at a regulatory level, of the events that give rise to new species or the emergence of specific characteristics. The correlation analysis done in this present study showed that lncRNAs might play some role in the response of young oil palm plants to both drought and salinity stresses. Long non-coding RNAs (lncRNAs) are >200 nucleotides-long RNAs that are not translated into functional proteins but have cellular functions of a structural and/or regulatory nature (Statello et al., 2021). Accordingly to Jha and colleagues, lncRNAs play an essential role in plant adaptation to various abiotic stresses, such as drought, heat, cold, heavy metal toxicity, and nutrient deficiency (Jha et al., 2020).

The Major Facilitator Superfamily (MFS) is the largest group of secondary active membrane transporters that shows a much larger number of genes in plant genomes than in bacteria, yeast, or animals (Niño-González et al., 2019). Again, the correlation analysis done in this present study showed that an MFS gene plays a role in the response of young oil palm plants to both abiotic stresses under consideration. Genes from the MFS are involved in plant response to abiotic stress in arabidopsis, mediating drought and salt tolerance (Remy et al., 2013; Wang, D. et al., 2020).

Finally, regarding the remaining three proteins shown to be potentially playing a role in the response of young oil palm plant to drought and salinity stresses – a putative histone-modifying enzyme harboring a demethylase activity, a protein from the class III homeodomain-leucine zipper family, and a protein from the MIF family, reports are there linking them to stress memory (Bhadouriya et al., 2021), regulation of defense response to abiotic and biotic stresses (Sharif et al., 2021), and modulation of ROS signaling (Zhao et al., 2021).

Many studies are available where the main objective is to compare the effects of those or more two types of stresses on plant growth and development (Chaves et al., 2009; de Oliveira et al., 2013; Uddin et al., 2016; Ma et al., 2020). This present study allowed us to visualize some of the similarities and dissimilarities – regarding expression analysis of miRNAs and their putative target genes – young oil palm plants have with the response to two highly important environmental stresses, drought and salinity. As far as we know, this is the first time such type of study is done in oil palm.

The present study and our previous one (Salgado et al., 2021) used plants with the same genetic background (clones from the same plant), the same age (young oil palm plants), almost similar duration of stress (12 and 14 days, respectively for salinity and drought stress), same omics platform (transcriptome of mRNAs and smallRNAs), and the same group of analytical tools. All these commonalities between these two studies allowed us analyze the similarities and dissimilarities regarding expression analysis of miRNAs and their putative target genes, what it a valuable set of information to help us in the search for genes that can be used to promote future attempts to horizontally transfer tolerance at once to both stresses; not only to oil palm, but also other plant species (Patel et al., 2019; Chaudhary et al., 2021).

## Conclusion

This study characterized the miRNA population and their miRNA-target genes present in the leaves of young oil palm plants exposed to drought stress, besides it performed correlation analysis of the miRNAs and their target genes differentially expressed under drought and salinity stresses (from Salgado et al., 2021). Together, those activities resulted in:

- The identification of two new miRNAs that received the names egu-miR28ds and egu-miR29ds, where egu is the abbreviation of *Elaeis guineensis* and ds stands for drought stress;
- The prediction of 185 distinct genes as the targets to the 81 miRNAs in the genome of oil palm and a total of 102 miRNA-mRNA interactions involving 62 DE miRNAs and 88 DE mRNAs;
- Among the 88 DE target genes selected for functional annotation, 32 had positive hits for biological processes, 50 for molecular function, and 19 for cellular components; and.
- Eight miRNA-putative target genes – upregulated under salinity as well as drought stress – that code for lncRNAs, proteins from the Major facilitator superfamily, a putative histone-modifying enzyme harboring a demethylase activity, a protein from the class III homeodomain-leucine zipper family, and a protein from the Macrophage migration inhibitory factor (MIF) family.

## Data availability statement

The datasets presented in this study can be found in online repositories. The names of the repository/repositories and accession number(s) can be found at: https://www.ncbi.nlm.nih.gov/, The smallRNA raw sequence data used in this study have been uploaded in the SRA database of the NCBI under Elaeis guineensis microRNA_Drought and Salinity Stresses – BioProject PRJNA646488 (SUB7775347), BioSample SAMN12799239 (SUB6325749), SRA submission SUB7897143 (accessions from SRR12424937 to SRR12424945). All RNA-seq fastq files used in this study have been uploaded in the SRA database of the NCBI under Elaeis guineensis Transcriptome_Drought and Salinity Stresses – BioProject PRJNA573093 (SUB6324604), BioSample SAMN12799239 (SUB6325749), SRA submission SUB6335775 (accessions from SRR10219424 to SRR10219441). The data-sets used and/or analyzed during the current study are available from the corresponding author on reasonable request.

## Author contributions

CS and MS designed the study. FS, TS, LV, VS, and AL performed the experiments and generated the data. FS, PG, MC, RT, CS, and MS analyzed the data. FS, PG, CS, and MS wrote first draft of the manuscript, which was extensively edited and approved the submitted version by all authors. MS was responsible for the funding acquisition, project administration, and group supervision. All authors contributed to the article and approved the submitted version.

## Funding

The grant (01.13.0315.00—DendePalm Project) for this study was awarded by the Brazilian Ministry of Science, Technology, and Innovation (MCTI) *via* the Brazilian Research and Innovation Agency (FINEP). The authors confirm that the funder had no influence over the study design, the content of the article, or the selection of this journal.

## Conflict of interest

AL, MC, RT, CS, PG, and MS were employed by The Brazilian Agricultural Research Corporation.

The remaining authors declare that the research was conducted in the absence of any commercial or financial relationships that could be construed as a potential conflict of interest.

## Publisher’s note

All claims expressed in this article are solely those of the authors and do not necessarily represent those of their affiliated organizations, or those of the publisher, the editors and the reviewers. Any product that may be evaluated in this article, or claim that may be made by its manufacturer, is not guaranteed or endorsed by the publisher.

## Footnotes

^1^ www.asd-cr.com

^2^ https://sourceforge.net/projects/mireap

^3^ https://github.com/MikeAxtell/ShortStack

^4^ https://github.com/MikeAxtell/strucVis

^5^ http://www.mirbase.org/search.shtml

^6^ https://bio.tools/psrnatarget

^7^ https://fusion.cebitec.uni-bielefeld.de

^8^ www.github.com/MikeAxtell/strucVis

^9^ www.mirbase.org

^10^ www.bio.tools/psrnatarget

^11^ https://cytoscape.org

## References

[ref1] Abrapalma (2018). Retrospecto e Projeções da Palma de Óleo No Brasil. Available at: http://www.abrapalma.org/pt/retrospecto-e-projecoes-da-palma-de-oleo-no-brasil-2018-2019/.

[ref2] Anders S. Pyl P. T. Huber W. (2015). HTSeq–a python framework to work with high-throughput sequencing data. Bioinformatics 31, 166–169. doi: 10.1093/bioinformatics/btu638, PMID: 25260700 PMC4287950

[ref3] Andrews S. (2018). FastQC: A quality control tool for high Thoughput sequence 2010. Data. Available at: from https://www.bioinformatics.babraham.ac.uk/projects/fastqc/ (Retrieved 2018).

[ref4] Axtell M. J. Meyers B. C. (2018). Revisiting criteria for plant micro RNA annotation in the era of big data. Plant Cell 30, 272–284. doi: 10.1105/tpc.17.00851, PMID: 29343505 PMC5868703

[ref5] Azzeme A. M. Abdullah S. N. A. Aziz M. A. Wahab P. E. M. (2016). Oil palm leaves and roots differ in physiological response, antioxidant enzyme activities and expression of stress-responsive genes upon exposure to drought stress. Acta Physiol. Plant. 38:52. doi: 10.1007/s11738-016-2073-2

[ref6] Bhadouriya S. L. Mehrotra S. Basantani M. K. Loake G. J. Mehrotra R. (2021). Role of chromatin architecture in plant stress responses: an update. Front. Plant Sci. 11:603380. doi: 10.3389/fpls.2020.603380, PMID: 33510748 PMC7835326

[ref7] Bolger A. M. Lohse M. Usadel B. (2014). Trimmomatic: a flexible trimmer for Illumina sequence data. Bioinformatics 30, 2114–2120. doi: 10.1093/bioinformatics/btu170, PMID: 24695404 PMC4103590

[ref001] Brink B. G. Seidel A. Kleinbölting N. Nattkemper T. W. Albaum S. P. (2016). Omics fusion – a platform for integrative analysis of omics data. J. Integr. Bioinform. 13, 43–46. doi: 10.1515/jib-2016-296 28187412

[ref8] Cantalapiedra C. P. Hernández-Plaza A. Letunic I. Bork P. Huerta-Cepas J. (2021). eggNOG-mapper v2: functional annotation, Orthology assignments, and domain prediction at the metagenomic scale. Mol. Biol. Evol. 38, 5825–5829. doi: 10.1093/molbev/msab293, PMID: 34597405 PMC8662613

[ref9] Chaudhary S. Grover A. Sharma P. C. (2021). MicroRNAs: potential targets for developing stress-tolerant crops. Life (Basel, Switzerland) 11:289. doi: 10.3390/life11040289, PMID: 33800690 PMC8066829

[ref10] Chaves M. M. Flexas J. Pinheiro C. (2009). Photosynthesis under drought and salt stress: regulation mechanisms from whole plant to cell. Ann. Bot. 103, 551–560. doi: 10.1093/aob/mcn125, PMID: 18662937 PMC2707345

[ref11] Chen X. (2004). A MicroRNA as a translational repressor of APETALA2 in Arabidopsis flower development. Science 303, 2022–2025. doi: 10.1126/science.1088060, PMID: 12893888 PMC5127708

[ref12] Chen G. Zou Y. Hu J. Ding Y. (2018). Genome-wide analysis of the Rice PPR gene family and their expression profiles under different stress treatments. BMC Genomics 19:720. doi: 10.1186/s12864-018-5088-9, PMID: 30285603 PMC6167770

[ref13] Comai L. Zhang B. (2012). MicroRNAs: key gene regulators with versatile functions. Plant Mol. Biol. 80:1. doi: 10.1007/s11103-012-9947-5, PMID: 22825768

[ref14] Corley R. H. V. (2009). How much palm oil do we need? Environ. Sci. Pol. 12, 134–139. doi: 10.1016/j.envsci.2008.10.011

[ref15] Corley R. H. V. Rao V. Palat T. Praiwan T. (2018). Breeding for drought tolerance in oil palm. J. Oil Palm Res. 30, 26–35.

[ref16] Corrêa T. R. Motoike S. Y. Coser S. M. DA Silveira G. De Resende M. D. V. Chia G. S. (2015). Estimation of genetic parameters for *in vitro* oil palm characteristics (*Elaeis guineensis* Jacq.) and selection of genotypes for cloning capacity and oil yield. Ind. Crop. Prod. 77, 1033–1038. doi: 10.1016/j.indcrop.2015.09.066

[ref17] Cutler S. R. Rodriguez P. L. Finkelstein R. R. Abrams S. R. (2010). Abscisic acid: emergence of a Core signaling network. Annu. Ver. Plant Biol 61, 651–679. doi: 10.1146/annurev-arplant-042809-112122, PMID: 20192755

[ref18] da Silva A. C. Grativol C. Thiebaut F. Hemerly A. S. Ferreira P. C. (2016). Computational identification and comparative analysis of miRNA precursors in three palm species. Planta 243, 1265–1277. doi: 10.1007/s00425-016-2486-6, PMID: 26919984

[ref19] de Oliveira A. B. Alencar N. L. M. Gomes-Filho E. (2013). “Comparison between the water and salt stress effects on plant growth and development,” in Responses of Organisms to Water Stress (IntechOpen)

[ref20] Denli A. M. Tops B. B. J. Plasterk R. H. A. Ketting R. F. Hannon G. J. (2004). Processing of primary microRNAs by the microprocessor complex. Nature 432, 231–235. doi: 10.1038/nature03049 15531879

[ref21] Ding D. Zhang L. Wang H. Liu Z. Zhang Z. Zheng Y. (2009). Differential expression of miRNAs in response to salt stress in maize roots. Ann. Bot. 103, 29–38. doi: 10.1093/aob/mcn205, PMID: 18952624 PMC2707283

[ref22] Djami-Tchatchou A. T. Sanan-Mishra N. Ntushelo K. Dubery I. A. (2017). Functional roles of microRNAs in Agronomically important plants-potential as targets for crop improvement and protection. Front. Plant Sci. 8:378. doi: 10.3389/fpls.2017.00378, PMID: 28382044 PMC5360763

[ref23] Dobin A. Davis C. A. Schlesinger F. Drenkow J. Zaleski C. Jha S.. (2013). STAR: ultrafast universal RNA-seq aligner. Bioinformatics 29, 15–21. doi: 10.1093/bioinformatics/bts635, PMID: 23104886 PMC3530905

[ref24] EPOA (2020). Palm oil production. Available at: https://www.palmoilandfood.eu/en/palm-oil-production

[ref25] Fang L. Liang Y. Li D. Cao X. Zheng Y. (2013). Dynamic expression analysis of miRNAs during the development process of oil palm mesocarp. Plant Sci. 31, 304–312. doi: 10.3724/SP.J.1142.2013.30304

[ref26] Flexas J. Barbour M. M. Brendel O. Cabrera H. M. Carriquí M. Díaz-Espejo A.. (2012). Corrigendum to ‘mesophyll diffusion conductance to CO2: an unappreciated central player in photosynthesis. Plant Sci. 196:31. doi: 10.1016/j.plantsci.2012.08.001 22794920

[ref27] Gandikota M. Birkenbihl R. P. Hohmann S. Cardon G. H. Saedler H. Huijser P. (2007). The miRNA156/157 recognition element in the 3′ UTR of the Arabidopsis SBP box gene SPL3 prevents early flowering by translational inhibition in seedlings. Plant J. 49, 683–693. doi: 10.1111/j.1365-313X.2006.02983.x, PMID: 17217458

[ref28] Gray-Mitsumune M. Matton D. P. (2006). The egg apparatus 1 gene from maize is a member of a large gene family found in both monocots and dicots. Planta 223, 618–625. doi: 10.1007/s00425-005-0174-z, PMID: 16341706

[ref29] Ho H. Gudimella R. Ong-Abdullah M. Harikrishna J. A. (2017). Expression of microRNAs during female inflorescence development in African oil palm (*Elaeis guineensis* Jacq.). Tree Genet. Genomes 13:35. doi: 10.1007/s11295-017-1120-5

[ref30] Hou H. Jia H. Yan Q. Wang X. (2018). Overexpression of a SBP-box gene (VpSBP16) from Chinese wild Vitis species in Arabidopsis improves salinity and drought stress tolerance. Int. J. Mol. Sci. 19:940. doi: 10.3390/ijms19040940, PMID: 29565279 PMC5979544

[ref31] Huerta-Cepas J. Szklarczyk D. Heller D. Hernández-Plaza A. Forslund S. K. Cook H.. (2019). eggNOG 5.0: a hierarchical, functionally and phylogenetically annotated orthology resource based on 5090 organisms and 2502 viruses. Nucleic Acids Res. 47, D309–D314. doi: 10.1093/nar/gky1085, PMID: 30418610 PMC6324079

[ref32] Jha U. C. Nayyar H. Jha R. Khurshid M. Zhou M. Mantri N.. (2020). Long non-coding RNAs: emerging players regulating plant abiotic stress response and adaptation. BMC Plant Biol. 20:466. doi: 10.1186/s12870-020-02595-x, PMID: 33046001 PMC7549229

[ref33] Kanehisa M. Sato Y. Morishima K. (2016). BlastKOALA and GhostKOALA: KEGG tools for functional characterization of genome and Metagenome sequences. J. Mol. Biol. 428, 726–731. doi: 10.1016/j.jmb.2015.11.006, PMID: 26585406

[ref34] Langmead B. Trapnell C. Pop M. Salzberg S. L. (2009). Ultrafast and memory-efficient alignment of short DNA sequences to the human genome. Genome Biol. 10:R25. doi: 10.1186/gb-2009-10-3-r25, PMID: 19261174 PMC2690996

[ref35] Li W. X. Oono Y. Zhu J. He X. J. Wu J. M. Lida K.. (2008). The Arabidopsis NFYA5 transcription factor is regulated transcriptionally and Posttranscriptionally to promote drought resistance. Plant Cell 20, 2238–2251. doi: 10.1105/tpc.108.059444, PMID: 18682547 PMC2553615

[ref36] Liu H. H. Tian X. Li Y. J. Wu C. A. Zheng C. C. (2008). Microarray-based analysis of stress-regulated microRNAs in *Arabidopsis thaliana*. RNA 14, 836–843. doi: 10.1261/rna.895308, PMID: 18356539 PMC2327369

[ref37] Lopes Filho W. R. L. Rodrigues F. H. S. Ferreira I. V. L. Correa L. O. Cunha R. L. Pinheiro H. A. (2021). Physiological responses of young oil palm (*Elaeis guineensis* Jacq.) plants to repetitive water deficit events. Ind. Crop. Prod. 172:114052. doi: 10.1016/j.indcrop.2021.114052

[ref38] Low E. T. Rosli R. Jayanthi N. Mohd-Amin A. H. Azizi N. Chan K. L.. (2014). Analyses of hypomethylated oil palm gene space. PLoS One 9:e86728. doi: 10.1371/journal.pone.0086728, PMID: 24497974 PMC3907425

[ref39] Lu S. Sun Y. H. Chiang V. L. (2008). Stress-responsive microRNAs in Populus. Plant J. 55, 131–151. doi: 10.1111/j.1365-313X.2008.03497.x, PMID: 18363789

[ref40] Lu S. Sun Y. H. Clark C. Li L. Chiang V. L. (2005). Novel and mechanical stress-responsive MicroRNAs in *Populus trichocarpa* that are absent from Arabidopsis. Plant Cell 17, 2186–2203. doi: 10.1105/tpc.105.033456, PMID: 15994906 PMC1182482

[ref41] Lv D. K. Bai X. Li Y. Ding X. D. Ge Y. Cai H.. (2010). Profiling of cold-stress-responsive miRNAs in Rice by microarrays. Gene 459, 39–47. doi: 10.1016/j.gene.2010.03.011, PMID: 20350593

[ref42] Lytle J. R. Yario T. A. Steitz J. A. (2007). Target mRNAs are repressed as efficiently by microRNA-binding sites in the 5′ UTR as in the 3′ UTR. Proc. Natl. Acad. Sci. U. S. A. 104, 9667–9672. doi: 10.1073/pnas.0703820104, PMID: 17535905 PMC1887587

[ref43] Ma Y. Dias M. C. Freitas H. (2020). Drought and salinity stress responses and microbe-induced tolerance in plants. Front. Plant Sci. 11:591911. doi: 10.3389/fpls.2020.591911, PMID: 33281852 PMC7691295

[ref44] Mallory A. C. Reinhart B. J. Jones-Rhoades M. W. Tang G. Zamore P. D. Barton M. K.. (2004). MicroRNA control of PHABULOSA in leaf development: importance of pairing to the microRNA 5′ region. EMBO J. 23, 3356–3364. doi: 10.1038/sj.emboj.7600340, PMID: 15282547 PMC514513

[ref45] Mantovani R. (1999). The molecular biology of the CCAAT-binding factor NF-Y. Gene 239, 15–27. doi: 10.1016/s0378-1119(99)00368-6 10571030

[ref46] Martin M. (2011). Cutadapt removes adapter sequences from high-throughput sequencing reads. EMBnet J, 17, 10–12. ISSN 2226–6089. Available at: http://journal.embnet.org/index.php/embnetjournal/article/view/200

[ref47] Md Nasaruddin N. Harikrishna K. Othman R. Hoon L. Ann Harikrishna J. (2007). Computational prediction of microRNAs from oil palm (*Elaeis guineensis Jacq*.) expressed sequence tags. Asia Pac. J. Mol. Biol. Biotechnol. 15, 107–113.

[ref48] Meerow A. W. Krueger R. R. Singh R. Low E. T. L. Ithnin M. Ooi L. C. L. (2012). “Coconut, date, and oil palm genomics,” in Genomics of Tree Crops. eds. Schnell R. Priyadarshan P. (New York, NY: Springer).

[ref49] Nelson D. E. Repetti P. P. Adams T. R. Creelman R. A. Wu J. Warner D. C.. (2007). Plant nuclear factor Y (NF-Y) B subunits confer drought tolerance and Lead to improved corn yields on water-limited acres. Proc. Natl. Acad. Sci. U. S. A. 104, 16450–16455. doi: 10.1073/pnas.0707193104, PMID: 17923671 PMC2034233

[ref50] Niño-González M. Novo-Uzal E. Richardson D. N. Barros P. M. Duque P. (2019). More transporters, more substrates: the Arabidopsis Major facilitator superfamily revisited. Mol. Plant 12, 1182–1202. doi: 10.1016/j.molp.2019.07.003, PMID: 31330327

[ref51] OmicsBox (2019). Bioinformatics made easy, BioBam bioinformatics, March 3. Available at: https://www.biobam.com/omicsbox

[ref52] Patel P. Yadav K. Ganapathi T. R. Penna S. (2019). “Plant miRNAome: cross talk in abiotic stressful times,” in Genetic enhancement of crops for tolerance to abiotic stress: Mechanisms and approaches, Vol. I. Sustainable Development and Biodiversity. eds. *vol. 20* Rajpal V. Sehgal D. Kumar A. Raina S., (Cham: Springer).

[ref53] Qiu C.-W. Liu L. Feng X. Hao P.-F. He X. Cao F.. (2020). Genome-wide identification and characterization of drought stress responsive microRNAs in Tibetan wild barley. Int. J. Mol. Sci. 21:2795. doi: 10.3390/ijms21082795, PMID: 32316632 PMC7216285

[ref54] Remy E. Cabrito T. R. Baster P. Batista R. A. Teixeira M. C. Friml J.. (2013). A major facilitator superfamily transporter plays a dual role in polar auxin transport and drought stress tolerance in Arabidopsis. The Plant cell vol. 25, 901–926. doi: 10.1105/tpc.113.110353, PMID: 23524662 PMC3634696

[ref56] Salgado F. F. Vieira L. R. Silva V. Leão A. P. Grynberg P. do Carmo Costa M. M.. (2021). Expression analysis of miRNAs and their putative target genes confirm a preponderant role of transcription factors in the early response of oil palm plants to salinity stress. BMC Plant Biol. 21:518. doi: 10.1186/s12870-021-03296-9, PMID: 34749653 PMC8573918

[ref57] Sharif R. Raza A. Chen P. Li Y. el-Ballat E. M. Rauf A.. (2021). HD-ZIP gene family: potential roles in improving plant growth and regulating stress-responsive mechanisms in plants. Genes 12:1256. doi: 10.3390/genes12081256, PMID: 34440430 PMC8394574

[ref58] Silva P. A. Cosme V. S. Rodrigues K. C. B. Detmann K. S. C. Leão F. M. Cunha R. L.. (2017). Drought tolerance in two oil palm hybrids as related to adjustments in carbon metabolism and vegetative growth. Acta Physiol. Plant. 39:58. doi: 10.1007/s11738-017-2354-4

[ref59] Silva P. A. Oliveira I. V. Rodrigues K. C. B. Cosme V. S. Bastos A. J. R. Detmann K. S. C.. (2016). Leaf gas exchange and multiple enzymatic and non-enzymatic antioxidant strategies related to drought tolerance in two oil palm hybrids. Trees – Struct. Function 30, 203–214. doi: 10.1007/s00468-015-1289-x

[ref60] Singh R. Ong-Abdullah M. Low E. T. Manaf M. A. Rosli R. Nookiah R.. (2013). Oil palm genome sequence reveals divergence of interfertile species in old and new worlds. Nature 500, 335–339. doi: 10.1038/nature12309, PMID: 23883927 PMC3929164

[ref61] Statello L. Guo C. J. Chen L. L. Huarte M. (2021). Gene regulation by long non-coding RNAs and its biological functions. Nat. Rev. Mol. Cell Biol. 22, 96–118. doi: 10.1038/s41580-020-00315-9, PMID: 33353982 PMC7754182

[ref62] Sun G. (2012). MicroRNAs and their diverse functions in plants. Plant Mol. Biol. 80, 17–36. doi: 10.1007/s11103-011-9817-6 21874378

[ref63] Sunkar R. Girke T. Jain P. K. Zhu J. K. (2005). Cloning and characterization of microRNAs from Rice. Plant Cell 17, 1397–1411. doi: 10.1105/tpc.105.031682, PMID: 15805478 PMC1091763

[ref64] Tarazona S. Furió-Tarí P. Turrà D. Pietro A. D. Nueda M. J. Ferrer A.. (2015). Data quality aware analysis of differential expression in RNA-seq with NOISeq R/bioc package. Nucleic Acids Res. 43:e140. doi: 10.1093/nar/gkv711, PMID: 26184878 PMC4666377

[ref65] Trindade I. Capitão C. Dalmay T. Fevereiro M. P. dos Santos D. M. (2010). miR398 and miR408 are up-regulated in response to water deficit in *Medicago truncatula*. Planta 231, 705–716. doi: 10.1007/s00425-009-1078-0, PMID: 20012085

[ref66] Uddin M. N. Hossain M. A. Burritt D. (2016). “Salinity and drought stress: similarities and differences in oxidative responses and cellular redox regulation” in Water Stress and Crop Plants: A Sustainable Approach. ed. Ahmad P. (Hoboken, NJ: Wiley), 86–101.

[ref67] Vaucheret H. Vazquez F. Crété P. Bartel D. P. (2004). The action of ARGONAUTE1 in the miRNA pathway and its regulation by the miRNA pathway are crucial for plant development. Genes Dev. 18, 1187–1197. doi: 10.1101/gad.1201404, PMID: 15131082 PMC415643

[ref68] Ventura A. Young A. G. Winslow M. M. Lintault L. Meissner A. Erkeland S. J.. (2008). Targeted deletion reveals essential and overlapping functions of the miR-17∼92 family of miRNA clusters. Cells 132, 875–886. doi: 10.1016/j.cell.2008.02.019, PMID: 18329372 PMC2323338

[ref69] Wang T. Chen L. Zhao M. Tian Q. Zhang W. H. (2011). Identification of drought-responsive microRNAs in *Medicago truncatula* by genome-wide high-throughput sequencing. BMC Genomics 12:367. doi: 10.1186/1471-2164-12-367, PMID: 21762498 PMC3160423

[ref70] Wang L. Lee M. Ye B. Yue G. H. (2020). Genes, pathways and networks responding to drought stress in oil palm roots. Sci. Rep. 10, 21303–21313. doi: 10.1038/s41598-020-78297-z, PMID: 33277563 PMC7719161

[ref71] Wang D. Liu H. Wang H. Zhang P. Shi C. (2020). A novel sucrose transporter gene *IbSUT4* involves in plant growth and response to abiotic stress through the ABF-dependent ABA signaling pathway in Sweetpotato. BMC Plant Biol. 20:157. doi: 10.1186/s12870-020-02382-8, PMID: 32293270 PMC7157994

[ref73] Wei L. Zhang D. Xiang F. Zhang Z. (2009). Differentially expressed miRNAs potentially involved in the regulation of defense mechanism to drought stress in maize seedlings. Int. J. Plant Sci. 170, 979–989. doi: 10.1086/605122

[ref74] Wu G. Poethig R. S. (2006). Temporal regulation of shoot development in *Arabidopsis thaliana* by miRr156 and its target SPL3. Development 133, 3539–3547. doi: 10.1242/dev.02521, PMID: 16914499 PMC1610107

[ref75] Xiao Y. Xia W. Yang Y. Mason A. S. Lei X. Ma Z. (2013). Characterization and evolution of conserved MicroRNA through duplication events in date palm (Phoenix Dactylifera). PLoS One 8:71435. doi: 10.1371/journal.pone.0071435, PMID: 23951162 PMC3738527

[ref76] Xin C. Liu W. Lin Q. Zhang X. Cui P. Li F.. (2015). Profiling microRNA expression during multi-staged date palm (*Phoenix dactylifera* L.) fruit development. Genomics 105, 242–251. doi: 10.1016/j.ygeno.2015.01.004, PMID: 25638647

[ref77] Xu J. Chen Q. Liu P. Jia W. Chen Z. Xu Z. (2019). Integration of mRNA and miRNA analysis reveals the molecular mechanism underlying salt and alkali stress tolerance in tobacco. Int. J. Mol. Sci. 20:2391. doi: 10.3390/ijms20102391, PMID: 31091777 PMC6566703

[ref78] Zeeshan M. Qiu C.-W. Naz S. Cao F. Wu F. (2021). Genome-wide discovery of miRNAs with differential expression patterns in responses to salinity in the two contrasting wheat cultivars. Int. J. Mol. Sci. 22:12556. doi: 10.3390/ijms222212556, PMID: 34830438 PMC8621374

[ref80] Zhao M. Chang Q. Liu Y. Sang P. Kang Z. Wang X. (2021). Functional characterization of the wheat macrophage migration inhibitory factor TaMIF1 in wheat-stripe rust (*Puccinia striiformis*) interaction. Biology 10:878. doi: 10.3390/biology10090878, PMID: 34571757 PMC8470491

[ref81] Zheng Y. Chen C. Liang Y. Sun R. Gao L. Liu T.. (2019). Genome-wide association analysis of the lipid and fatty acid metabolism regulatory network in the mesocarp of oil palm (*Elaeis guineensis* Jacq.) based on small noncoding RNA sequencing. Tree Physiol. 39, 356–371. doi: 10.1093/treephys/tpy091, PMID: 30137626

[ref82] Zhou L. Liu Y. Liu Z. Kong D. Duan M. Luo L. (2010). Genome-wide identification and analysis of drought-responsive microRNAs in *Oryza Sativa*. J. Exp. Bot. 61, 4157–4168. doi: 10.1093/jxb/erq237, PMID: 20729483

[ref83] Zhu Q. H. Upadhyaya N. M. Gubler F. Helliwell C. A. (2009). Over-expression of miR172 causes loss of spikelet determinacy and floral organ abnormalities in Rice (Oryza Sativa). BMC Plant Biol. 9:149. doi: 10.1186/1471-2229-9-149, PMID: 20017947 PMC2803185

